# A Treatise on Therapeutics and Pharmacology

**Published:** 1856-12

**Authors:** 


For sale by D. B. Cooke & Co. Lake Street, Chicago.

---

*A Treatise on Therapeutics and Pharmacology, or Materia Medica.* By George B. Wood, M.D. Late President of the American Medical Association; President of the College of Physicians of Philadelphia; Professor of the Theory and Practice of Medicine in the University of Pennsylvania; Senior Physician to the Pennsylvania Hospital, &c. In Two Volumes. Philadelphia, J. B. Lippincott & Co. London, Trubner & Co. 1856.

The above is the title to a new and very valuable work on an important branch of medical science. It consists of two volumes, containing over eight hundred pages each, printed on good type and paper, and substantially bound in calf. The author was for many years an eminent teacher of Materia Medica, and, in the present volumes, has given to the profession one of the most complete and valuable text-books to which the student can have access. Like the work, of the same author, on the "Practice of Medicine," this will speedily find its way into the hands of a great majority of the students and practitioners of this country.

